# A Global Media Analysis of the Impact of the COVID-19 Pandemic on Chicken Meat Food Systems: Key Vulnerabilities and Opportunities for Building Resilience

**DOI:** 10.3390/su13169435

**Published:** 2021-08-23

**Authors:** Lorraine Chapot, Louise Whatford, Polly Compston, Mehroosh Tak, Soledad Cuevas, Maria Garza, Houda Bennani, Hassaan Bin Aslam, Mathew Hennessey, Georgina Limon, Kevin Queenan, Guillaume Fournié, Nikolaos Dadios, Barbara Häsler

**Affiliations:** 1 Veterinary Epidemiology, Economics and Public Health Group, Department of Pathobiology and Population Sciences, Royal Veterinary College, Hawkshead Lane, North Mymms, Hatfield, Hertfordshire AL9 7TA, UK; 2 Centre for Applied One Health Research and Policy Advice, City University of Hong Kong, 83 Tat Chee Avenue, Kowloon Tong, Hong Kong; 3 Department of Population Health, Faculty of Epidemiology and Population Health, London School of Hygiene and Tropical Medicine, Keppel Street, London WC1E 7HT, UK; 4 Department of Transmission Biology, The Pirbright Institute, Ash Road, Pirbright, Woking GU24 0NF, UK; 5 Institute of Microbiology, University of Veterinary and Animal Sciences, Out Fall Road, Lahore 54000, Pakistan

**Keywords:** food systems, COVID-19, chicken, media, systematic review

## Abstract

Food systems are increasingly under threat, with climate, biological, economic or policy shocks and stressors occurring at an increasing frequency and scale. Their complex and fragile nature has become even more apparent during the COVID-19 pandemic. A systematic review of news articles published globally between December 2019 and April 2020 was conducted to describe the impacts of the COVID-19 pandemic on the chicken meat system and to identify key vulnerabilities and intervention points to build resilience. Most of the impacts identified were caused by a combination of the different mitigation measures implemented by the system actors such as movement restrictions rather than by the direct effects of the virus, thereby demonstrating the importance of interconnections and coordination in the system. Social media were found to have played a crucial role in amplifying, mitigating or mediating the impact of the pandemic. The findings highlight the importance of adopting a holistic approach that integrates the multiple dimensions of food systems for effective responses to systemic shocks.

## 1 Introduction

Food systems consist of dynamic and complex networks formed by all people, infrastructures and processes involved in the production, distribution and consumption of food and the relationships between them [1–5]. The multiple connections among their different components are dynamic and often involve complex feedback loops that can be either reinforcing or disruptive [6]. Any change to these dynamics has the potential to alter the system balance and threaten food security or other desired food system outcomes. Capturing the diverse components of a system and understanding the continuously evolving interactions between them becomes particularly crucial when dealing with system-wide shocks such as climate change, natural disasters, economic hardship or, as illustrated by the COVID-19 pandemic, a health crisis [7].

The highly complex nature of food systems has become evident in the context of the current pandemic. In many countries, the consequences of mitigation measures, combined with the direct effects of the pandemic, have strongly impacted food systems and revealed their fragility in the face of disruptions. Restrictions of movements, closures of key businesses and changes in consumer behaviour have disrupted supply chains at multiple levels, raising concern over food security [3,8–10].

Being an affordable source of protein and accepted among most cultures and religions, chicken is the most consumed meat worldwide and plays an important role in sustaining the livelihoods and food security of many low- and middle-income households [8]. Chicken production and distribution networks involve a large diversity of actors including subsistence producers, smallholders, emerging producers and large integrated companies. Operations take place on a continuum spanning fully local systems and global networks for the acquisition of inputs (e.g., genetics, feed) or distribution of products. The respective nature of the operation is mirrored in the supporting governance, health services and finance flows. Therefore, the chicken meat food system was used in this study as a lens to investigate how food systems react to shocks.

Compared to the scientific literature, which often involves a lengthier publication process, public media aim to be at the pulse of events and generate real-time information about what is happening at a given place. They can provide direct and in-depth insights into the way the impacts of the COVID-19 pandemic are perceived and managed by the public [11]. As opposed to scientific publications in which the data are analysed and curated, the information provided by media retains all the context specificities and therefore offers a unique perspective of local dynamics and how they influence the wider system.

In this study, a systematic analysis of media reports was conducted to describe, map and analyse the diverse impacts of COVID-19 at the beginning of the pandemic on the global chicken meat system. Drawing from these insights, key vulnerabilities and opportunities for interventions to improve resilience were identified.

## 2 Materials and Methods

### 2.1 General Overview

A team of fourteen researchers was assembled in April 2020, with the aim of capturing the initial impacts of the pandemic on chicken food systems as reported through media. A content analysis of editorial media in English from 31 December 2019 to 24 April 2020 was conducted using guidance from the Preferred Reporting Items for Systematic Reviews and Meta-Analyses (PRISMA) as documented by Tricco et al. (2017) [12,13] and drawing upon similar studies [14–17]. This method applies a systematic approach to identify the presence and meanings of particular words or concepts in a qualitative dataset [18]. Details of the framework and its use in this study are further described in Section 2.2. Factiva, a search engine database that provides access to newspapers, television and radio transcripts was used to search for news that related simultaneously to COVID-19 and chickens (https://professional.dowjones.com/factiva/, accessed on 14 May 2021). Qualitative analysis and network visualisation were used to describe COVID-19-related changes to the chicken meat food system and to identify main themes and narratives. Opportunities for building resilience and entry points for interventions were then identified in an iterative process of collaborative narrative synthesis.

### 2.2 Search Strategy

An initial search was conducted in Factiva using a selection of search and exclusion terms (see supplementary Annex A). The cut-off date of 24 April was chosen to make sure that news outlets describing the impacts of the early stages of the pandemic would remain accessible and allow sufficient time to analyse the data, as news articles are sometimes available only for a short period of time. The terms were selected to ensure the search provided relevant articles, i.e., talked about any part of the chicken meat food system and COVID-19. Articles were included only if they contained at least twice the word “chicken” and/or “poultry” and/or at least one other poultry-related term (e.g., “hen,” “chick,” “fowl”), as they were considered more likely to be used in a relevant context (e.g., not in someone’s surname or as a saying). The Factiva search included any media reports published from 31 December 2019 (official start of the COVID-19 outbreak) to 24 April 2020 and automatically removed identical articles. Although no countries were excluded to allow for any narrative to emerge during the analysis, only articles in English were considered. Search terms were not translated, as this would have substantially complicated the search and delayed the analysis. All articles identified by the search in Factiva were downloaded chronologically, including a summary document and metadata (date of distribution and publisher name), and saved as PDF files in dated and numbered folders.

### 2.3 Content Inclusion and Exclusion

The research objective was to identify the different impacts of COVID-19 and mitigation strategies in chicken food systems to improve our understanding of the general principles governing food systems, identify key vulnerabilities and suggest interventions to improve resilience. A three-phase strategy was developed to assess the relevance of articles prior to data extraction through an interactive approach with team meetings once a week or once every fortnight.

#### 2.3.1 Preliminary Review

Each of the fourteen team members was allocated 15 articles for initial scanning by titles to reflect on the relevant inclusion criteria. Team members indicated in a Microsoft Excel spreadsheet whether or not the article seemed to be clearly relevant to the research question. Following initial scanning, the team members met to finalise inclusion/exclusion criteria and to develop a Microsoft Excel spreadsheet for initial data extraction. The final inclusion/exclusion criteria are presented in supplementary Annex B.

#### 2.3.2 Article Inclusion/Exclusion

Each team member was allocated between 300 and 600 articles to scan for relevance and reported the results of their screening in the Microsoft Excel spreadsheet developed in the preliminary review. Any article describing an impact of COVID-19 on the chicken meat production, distribution and consumption system was included. Articles where the words “chicken,” “poultry,” etc. were not related to animal or meat production (e.g., in a name, brand, as an ingredient or relating to egg production) or were not directly related to COVID-19 (e.g., articles compiling several unrelated news items) were excluded. Reviewers justified each decision by selecting one of the predefined exclusion criteria or specifying new criteria under the “Other” option. When the PDF document extracted from Factiva only included the title of the article, reviewers searched for the full article via a web browser and saved it as a PDF on a shared drive. Articles for which the relevance was deemed unclear were classified as potentially relevant and considered in the next stage of analysis.

#### 2.3.3 Quality Control

Following completion of the full review, 5% of the articles assigned to each researcher were randomly selected and reviewed by another team member to assess inter-rater reliability. Discrepancies were discussed among the two reviewers and during group meetings to homogenise the rationale for inclusion/exclusion decisions, to develop a common understanding among reviewers and to prepare for data extraction in the next stage.

### 2.4 Data Extraction, Categorization and Analysis

Data extraction and analysis were conducted following an iterative approach.

#### 2.4.1 Preliminary Review

Prior to the full extraction, each team member reviewed and extracted data from 15 articles to identify relevant themes and sub-themes. If they deemed the article as irrelevant, they would note the reason and suggest any specific terms that could be used to exclude similar articles. These were used to inform the development of an Excel extraction spreadsheet to standardise data collation. Themes and sub-themes that formed the column headings and subheadings in the spreadsheet were discussed and refined during group meetings to ensure that all aspects of media content were captured. Eight main themes were identified, which are detailed in supplementary Annex C: demand and market effects, value chain effects, trade, labour and livelihoods, mitigation and interventions, coping, epidemiological factors and animal welfare. For each sub-theme, extraction codes were listed in a dynamic drop-down menu to help standardise data extraction among reviewers.

#### 2.4.2 Information Extraction

Information from the dataset was extracted into an interactive data extraction spread-sheet in Microsoft Excel. For each article, reviewers reported the key information by selecting or creating a relevant extraction code in the drop-down menu in each sub-theme and including quotes from the articles. Articles that described the same narrative with minor variations, which prevented them from being originally identified as duplicates, were removed manually.

#### 2.4.3 Narrative Synthesis

An iterative, narrative synthesis was conducted by a sub-team of eleven researchers to report key findings regarding the effects of the pandemic on chicken meat systems and to explore relationships between its components. The total number of articles in each sub-theme and category are reported in supplementary Annex C. Prominent themes were identified based on their frequency and the results of network visualisation and were further discussed during seventeen group meetings. Countries for which we found less than 10 articles during the study period were not considered in the synthesis. Researchers whose dataset revolved around similar themes or geographical areas were grouped to discuss and perform a synthesis of their findings in the form of a narrative. This iterative and participatory process led to the description of key narratives relating to specific geographic areas or cross-regional topics.

## 3 Results

### 3.1 General Overview

**Figure 1:**
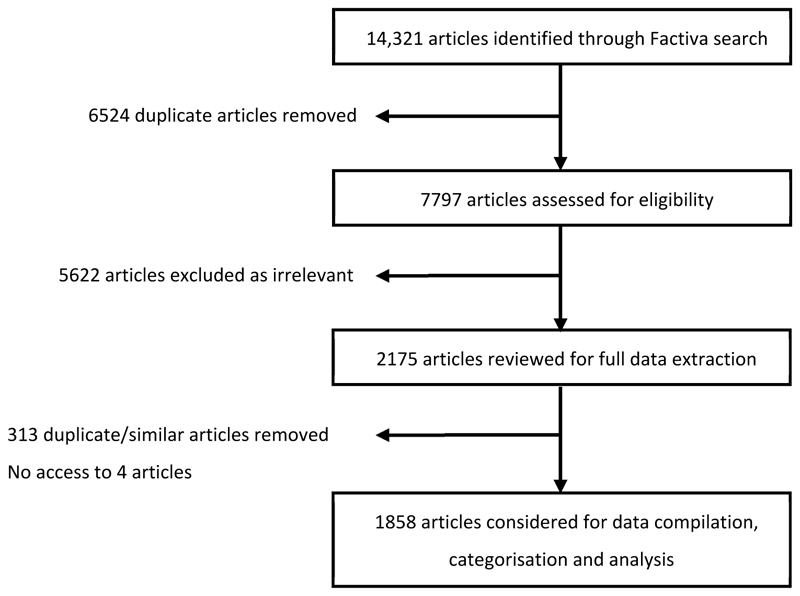
Flow chart documenting removal of duplicates and selection of articles for inclusion in the systematic review

**Figure 2:**
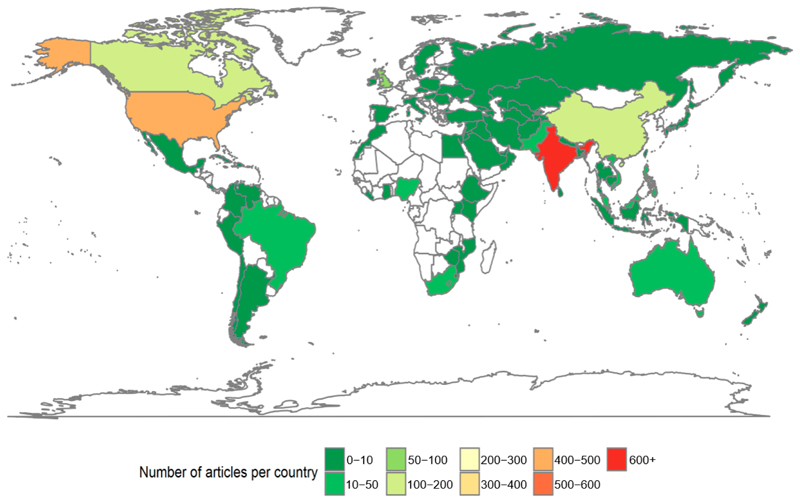
Number of articles per country in the final set of articles.

A total of 14,321 articles were identified from the search in Factiva, from which 6524 duplicates were automatically removed. After screening, 2175 articles were considered relevant for analysis. Three-hundred and thirteen additional duplicates were manually identified and removed (Figure 1), and 1858 articles were processed for full data extraction. Of the 195 sovereign nations, 70 were mentioned in the final selection, with India and the USA being the most frequent (Figure 2). The original dataset and the information extracted from the articles are available on the data repository RVC WorkTribe.

**Figure 3:**
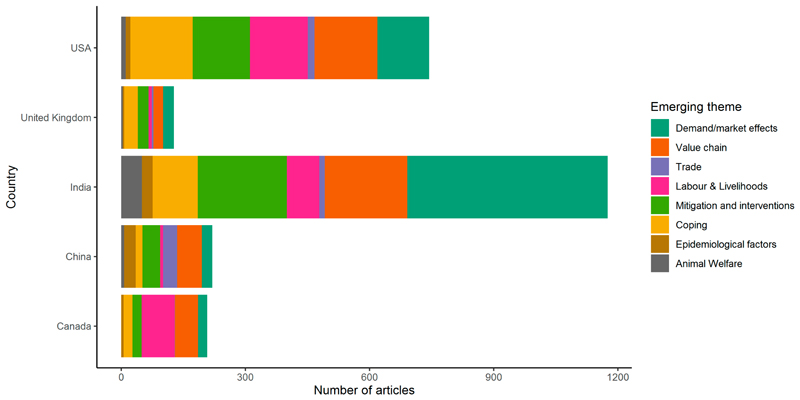
Example of emerging themes for five countries with >100 news articles reviewed.

**Figure 4:**
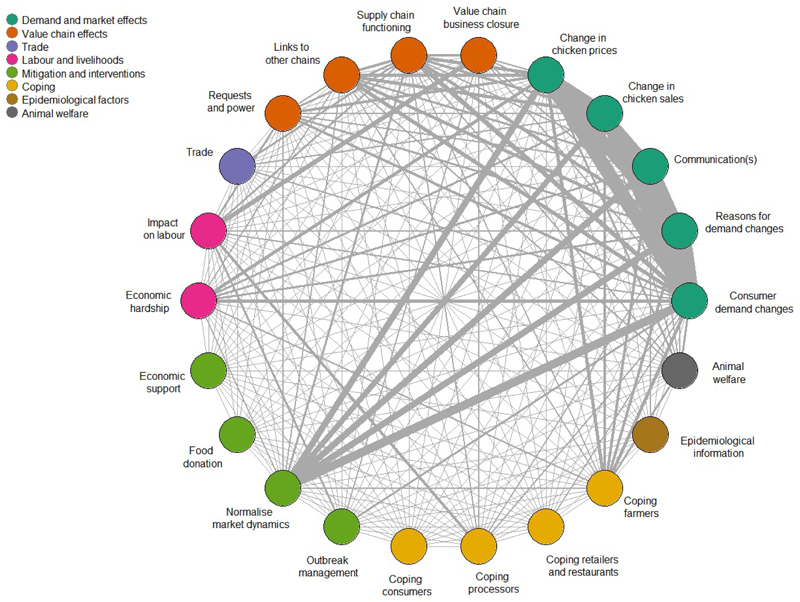
Network visualisation of connections between themes (legend) and sub-themes (circle).

After discussing the prominent themes emerging from the final set of articles (Figure 3), four major narratives relating to specific geographic areas or cross-regional themes were identified through an iterative participatory group process. Network visualisation illustrated the complex interconnections between themes and sub-themes (Figure 4). Due to the high number of articles and the qualitative nature of the data extraction process, it was not possible to include direct references to all sources that were used to frame the narratives.

### 3.2 Main Narratives

#### 3.2.1 National Market Disruptions

Disruptions to the supply chain resulting from COVID-19 mitigation measures were found to be a central theme across all geographical areas. There were multiple examples of local restrictions causing delays in the transit of poultry product, reducing access to markets and slaughterhouses and preventing migrant workers from travelling to their workplace. In the Philippines and China for instance, strict movement restrictions in quarantined areas blocked the delivery of farm inputs such as chicks or feed. Consequently, some farmers were forced to halt production and dispose of their birds, raising concerns over potential food shortages. These disruptions were considered to result from a lack of coordination between the government and police forces combined with a confusion about which “essential products” were exempted from lockdown restrictions. Diverse coping strategies were observed among the different system actors. Surpluses were often managed through the disposal of birds in India and China, whereas food donation was most common in high-income countries such as the USA. Policy makers also implemented various strategies to help farmers buy input and sell their products directly to consumers, for instance by setting up temporary trading centres or using online platforms offering farm-related products.

#### 3.2.2 International Trade and Dependencies

While disruptions were frequently described through a local lens, several reports highlighted their repercussions at the global level. As one of the top poultry producers and importers, the impact of the COVID-19 pandemic on the Chinese chicken system strongly affected international commodities markets due to a fear that local restrictions would weaken China’s demand. China’s ability to fulfil its commitment to increase purchases of USA agricultural goods was questioned, prompting USA chicken producers to divert exports to the domestic market. At the same time, the pandemic contributed to increase China’s dependency on overseas suppliers by adding to the strain on the domestic meat production system, which had already been weakened by recent outbreaks of Avian Influenza and African Swine Fever. In response, the Chinese government eased import restrictions to ensure sufficient meat supplies, creating new business opportunities as highlighted in several reports from exporting countries such as Brazil, the USA or Argentina. Likewise, the crisis revealed a strong reliance on foreign suppliers for chicken meat in Singapore, Mozambique and Afghanistan, with the closure of borders raising concern over potential food shortages.

#### 3.2.3 Changes in Consumer Demand: Panic Buying and Misinformation

The impact on demand for chicken meat varied across settings, with either sharp increases or decreases. In many countries, the closure of hospitality venues and schools resulted in a strong increase in demand for poultry products in the retail sector, which was further aggravated by panic buying as observed, for instance, in the UK, the USA and Singapore. In countries such as the USA where the system operates “just-in-time,” the switch from producing specifically for food services to retail created delays in supplies that led to empty store shelves, which further exacerbated fears.

In contrast, the majority of reports from India revolved around the spread of rumours on social media of COVID-19 being transmitted to humans through chicken meat, resulting in a sharp drop in demand. The subsequent decrease in prices was transmitted through-out the system, causing heavy income losses for restaurants, retailers, wholesalers and farmers. Multiple reports described farmers’ distress at not being able to sell their birds and resorting to mass culling to avoid the cost of keeping large unsold and unsellable stock. In efforts to normalise demand, government and industry stakeholders developed various communication strategies. There were numerous examples of events where chicken dishes were offered to the population or eaten by officials in public to allay fears. Underlying cultural values were highlighted, revealing tensions between differing ideologies: while proponents of vegetarianism blamed the “barbaric eating habits” of meat eaters and presented COVID-19 as a divine punishment, others considered chicken an essential component of a healthy diet that contributed to build immunity.

#### 3.2.4 Vulnerability of Workers

Outbreaks of COVID-19 in meat processing plants revealed the vulnerability of poultry chain workers and underlined their lack of social and financial support. Numerous articles from the USA reported stories of unwell employees feeling forced to attend work in fear of losing their source of income and voicing concerns over the lack of protective measures in plants. Articles also described the subsequent efforts of processors to adapt chain operations and work procedures to ensure the safety of workers (e.g., social distancing, testing and tracing of staff). The absence of government support was also apparent in India, threatening the livelihood of many smallholders who were suffering heavy economic losses.

## 4 Discussion

Media content analysis was used to describe the various impacts of the COVID-19 pandemic on chicken meat food systems and to provide an overview of major narratives. This method allowed reviewers to capture timely and unfiltered information that provided all the context specificities. Exploring those narratives led to the identification of key vulnerabilities of the system and intervention points for building resilience.

### 4.1 Key Vulnerabilities

#### 4.1.1 Interconnectedness of the Chicken Meat Food System

This analysis highlighted the complex interconnections and dependencies between the diverse actors of the system, which contribute both to its resilience and vulnerability. In China for instance, where disruptions to domestic market have impacted global trade dynamics and revealed a strong reliance on overseas suppliers, it also created new business opportunities for other exporting countries. Therefore, interconnectedness in a system can improve robustness to shocks impacting a single component or sub-system by providing opportunities for alternative actors to make up for the losses, also known as redundancy. However, it also increases the risk of concatenated shocks [6,7,19,20]. In other words, when one group is impacted, it is likely to trigger “ripple effects” affecting other parts of the system [4,6,21], which can lead to system-wide failure if no redundancy is built. This was commonly observed in areas under lockdown, where the effect of restrictions on the hospitality sector reverberated on multiple stakeholders from consumers to farmers. Adding to the complexity, the various responses of each actor to the shock can generate negative or positive feedback loops with often unpredictable consequences [21]. In line with other studies [3,10,21–23], we found that most of the impact of the COVID-19 pandemic on food systems was not related to the direct effects of the virus but to a combination of these effects with the various mitigation measures implemented by the different stakeholders. Most disruptions to the system were an adverse effect of movement restrictions that limited access to hired labour, inputs and informal retail outlets. In addition, detrimental coping strategies such as the culling of surplus birds or the closure of meat processing plants have contributed to the increased risk of food shortage and in some cases have triggered negative behaviours such as panic buying, which in turn aggravated the disruptions. It is therefore critical for policy makers to consider the effects of their response to the shock to avoid detrimental consequences [21]. This also underlines the need to acknowledge the political and socio-cultural dimensions of food systems [4,6].

Holistic approaches to food systems are needed to capture the complexity of these relationships and to ensure all constituents, interactions and outcomes are considered. As already emphasised by several studies, systems thinking can help anticipate the diverse impacts and feedback of the different systems components to prevent negative externalities and foster positive coping strategies (e.g., increasing the ability to find substitute workers or shift suppliers) [1–5,7,21,22,24–26].

#### 4.1.2 “Just-in-Time” Operations

Another key vulnerability identified was the “just-in-time” nature of some of the national food systems. Supply chains have been increasingly automated and streamlined to closely adjust production to demand and maximise performance. However, removing redundancy and diversity in a system can increase its fragility [4,20,27]. This was illustrated in the USA where labour shortage resulting from outbreaks of COVID-19 in automated processing plants halted the whole production system, while adaptations to the production lines to account for the shift in demand caused additional delays. In India, the absence of cold chain or storage capacity left farmers with no other alternative but to cull their unsold flocks to limit their losses. Although the race for profits has made it increasingly challenging, maintaining redundancy and diversity in a system is critical to help dealing with rapid change and to ensure continuity of supply when an area is failing.

#### 4.1.3 Power Imbalance

The definition and role of power in the value chains have been widely debated over many years. It has been described as a coercive concept where, for example, an actor uses rewards or penalties to mould others’ actions to achieve their own goal. However, power is also multi-dimensional and can be thought of as a relationship between two entities (e.g., farmers and processors) or bodies such as governments or social movements [28]. Advocates of collaborative approaches in food systems have argued that imbalanced power is a negative influence that impedes the formation of sustainable relationships by generating issues of dependencies and conflict, as opposed to cooperation. A contrasting viewpoint is that business relationships cannot develop solely on trust and that a form of power imbalance is not only inevitable but also a driving force necessary to the formation and operation of business partnerships [29]. Proponents of this opinion consider power imbalance as an inherent characteristic of value chains, as the value of the output generally exceeds that of the input. Therefore, relationships should not tend towards strict equality but rather should focus on combining and optimising individual contributions to achieve the common desired outcome [30]. However, our analysis has mostly highlighted some of the detrimental effects of power imbalance that arise when it exceeds the tolerance of the weaker parties. With the development of contract farming and vertical integration, modern food systems are increasingly dominated by a small number of large corporations. However, in many low- and middle-income countries poultry production also sustains the livelihood of many smallholders who are more vulnerable to shocks and often overlooked by policy makers [1,20,22]. Such an imbalance of power was apparent also in the USA where the crisis highlighted meat plants workers’ precarious situation.

Although not explicit, the imbalance between species appeared as an underlying issue. Chickens were commonly described as a commodity that could be disposed of when necessary, as illustrated by the numerous reports of mass culling across the globe. However, in some instances, they gave rise to public outcries and concerns over humane slaughter and created a rift between defenders of animal welfare and farmers who explained it as an act of desperation.

### 4.2 The Impact of Media

Our study demonstrated the active role of information flows in shaping policies and mediating the impact of the COVID-19 pandemic on food systems. As only articles in English were considered, our understanding of the various social and cultural specificities might have been limited. The inclusion of social media could also have offered a more in-depth insight into the local context and perceptions [11,18,30].

Having a strong influence on public opinion, attitudes and behaviours, media coverage has played an important role in framing the population response to the pandemic, thereby contributing to either mitigate or aggravate its impact [11,14,16,31]. In times of crisis, mass media are commonly used by policy makers to connect with the general public and reinforce adherence to mitigation measures [18]. In particular, the influence of the internet and social media has been growing in recent years but has so far received little attention in the literature [20,30]. Their impact on the chicken meat system was most striking in India where the spread of misinformation on social media about transmission of COVID-19 through chicken created a sharp drop in consumer demand and a collapse of prices from retail to farm level. Despite damage-control plans and official communication to address fears, stakeholders failed to restore confidence in the system and faced renewed criticism over poor production practices in the sub-sector.

The importance and challenges of effective communication and coordination for successful crisis management were further illustrated by numerous examples, notably in China and the Philippines where miscommunication from the authorities prevented the delivery of essential goods. Due to the large diversity of actors involved in the system, the creation of specific communication channels tailored to small networks of organisations in given geographic areas could facilitate coordination between and within sub-systems, thereby enabling further or future shocks to be managed more effectively [10,19]. Media can also play a valuable role in improving communication by allowing for the creation of feedback loops between stakeholders and policy makers [11]. In several geographical areas, media brought to light the particular vulnerability of poultry chain workers and the lack of consideration from policy makers. In the USA for instance, outbreaks of COVID-19 in meat processing plants gave workers the opportunity to initiate a debate around their poor working conditions and to request better social and financial government support. On another note, although explicit attribution of responsibility for the spread of the virus was scarce, the media framing around the origin of the virus contributed to reinforce misperceptions and spark an international outcry over China’s wet markets and production practices, which may have played a part in the Chinese government decision to legislate on wildlife trade.

## 5 Conclusions

The current pandemic can serve both as a warning and an opportunity to prepare for more frequent, simultaneous and cascading shocks in the future. From a theoretical perspective, this study has contributed to enhance our understanding of food systems by highlighting their strongly interconnected nature and how this contributes to both their fragility and resilience. These findings reinforce the necessity to anticipate potential ripple effects of control strategies to avoid unintended consequences. Additionally, while power relations have been described as a driving force, their detrimental influences were more apparent during the crisis. Drawing from those key vulnerabilities and learning from the diverse coping strategies described in this analysis, below are potential working principles to improve food system resilience in practice.

Firstly, as already emphasised by other studies [8,21,22], promoting inclusiveness is crucial to account for the diverse viewpoints of all contributing actors and ensure the weaker parties are not neglected. More collaborative and participatory approaches are required to engage all stakeholders in the negotiation and co-design of bundled interventions adapted to the socio-cultural context [4,25,32]. For example, the development of emergency policies or taskforces involving all actors of the food system, from smallholders to governments, would ensure procedures are in place to protect workers’ safety and rights, continued access to supplies and humane emergency animal slaughter procedures during any future shocks to the system.

Secondly, maintaining redundancy and diversity is essential to prevent the failure of a single node or link to propagate through the whole system [4,20,23,27]. Flexibility, which represents the capacity of the system to switch easily to an alternative, is also a critical feature in helping to cope with short-term disruptions [33]. For example, having more flexible local and international trade regulations would facilitate shifts to a larger range of suppliers and buyers. The COVID-19 crisis also emphasised the social and economic importance of non-human animals in sustaining human livelihoods, demonstrating that animal welfare and human wellbeing are interconnected issues. Therefore, integrating a “One Welfare” perspective in policy frameworks is likely to benefit both human and non-human animals [34].

Thirdly, shortening and diversifying supply chains have proven key to mitigate the impact of system shocks and to ensure access to food for the most vulnerable, for example, by facilitating direct purchases from producers as observed in several geographical areas during the crisis [1,10,21,22] or by reducing vertical integration to include a larger diversity of suppliers [30]. Achieving the “relocalisation” of food systems will require the development of diversified and innovative distribution channels to encourage new consumption habits and to empower producers. Digital technologies can provide interesting means to support these transformations, as shown by the use of online platforms to promote direct sales.

Finally, the role of media and communication must not be overlooked [11,20]. Timely monitoring of public media can help promote awareness of stakeholders’ views, develop social constitutive power and inform the development of more effective impact management strategies [14,18,30].

## Supplementary Material

Supplementary Materials: The following are available online at https://www.mdpi.com/article/10.3390/su13169435/s1, Annex A: Final search and exclusion terms, Annex B: Inclusion and exclusion criteria for review, Annex C: Final themes and codes used for data extraction.

Annex A

Annex B

Annex C

## Data Availability

The datasets generated in this study and the codes used to generate and analyse the results are available at https://rvc-repository.worktribe.com/output/1548769 (accessed on 14 May 2021).
