# Supplementary material for: A Global Media Analysis of the Impact of the COVID-19 Pandemic on Chicken Meat Food Systems: Key Vulnerabilities and Opportunities for Building Resilience
Source: Sustainability. Author manuscript; Available in PMC 2024 Dec 9. (PMC7617157; doi:10.3390/su13169435)
Supplement: Annex A [file EMS197569-supplement-Annex_A.pdf]

Annex A – Final search and exclusion terms

Factiva search terms used:

[[corona or coronavirus* or covid* or sars*] or [2019-ncov] or [sars-cov-2] or [corona w/1 virus]] AND[atleast2 chicken* or cock or cocks or chicks or chick or hen or hens or fowl or atleast2 poultry* or gallus* or [day w/1 old w/1 chick*]]

not [[chicken w/1 pox] or chickenpox or chickpea* or [chick w/1 pea]

or recipe* or joke* or sexy

or nrl or [national w/1 rugby w/1 league] or [burdekin w/1 rooster*] or [sydney w/1 rooster*] or [white w/1 rooster*] or [premier w/1 league] or [hei w/1 hei] or [omar w/1 asad] or [cruz w/1 azul] or [artemis w/1 fowl*] or fowle*

or dixie or [chick w/1 corea] or [tyson w/1 fury] or [martha w/1 stewart] or [side w/1 chick*]

or [hen w/1 do] or [hen w/1 dos] or [hen w/1 weekend*] or [hen w/1 part*] or [hen w/1 night*]

or cockfight* or [cock w/1 fight*] or [laying w/1 hen*]

or [the w/1 hen w/1 is w/1 killed w/1 to w/1 scare w/1 the w/1 monkey]

or chickenhawk or [yellow w/1 bellied w/1 chicken] or [speckled w/1 hen] or [gallus w/1 labelfire] or vetaphone

or kiwi or falcon or owl or ostrich

or sarson or sarsaparilla or sarso or sarsfield* or sarsgaard

or [corona w/1 sunsets] or [jesús w/1 corona] or beer* or [softal w/1 corona] or [joe w/1 corona] or [jo w/1 corona] or [ignacio w/1 corona]

]
