# Supplementary material for: A Global Media Analysis of the Impact of the COVID-19 Pandemic on Chicken Meat Food Systems: Key Vulnerabilities and Opportunities for Building Resilience
Source: Sustainability. Author manuscript; Available in PMC 2024 Dec 9. (PMC7617157; doi:10.3390/su13169435)
Supplement: Annex B [file EMS197569-supplement-Annex_B.pdf]

Annex B – Exclusion and inclusion criteria used for the review

**Exclusion criteria**
Use of words "Chicken", "Hen", etc unrelated to animals/food (e.g., surname, describe a person, brand name)
Use of words "Chicken", "Hen", etc that constitute a typo and do not relate to animals/food
Use of words "Chicken", "Hen", etc for recipe/ingredient/diet
Focus on eggs, not meat
Not related to COVID19 (not related to diseases at all, or to another one such as bird flu)
About restaurant(s) or retailer(s) that has/have not a poultry or chicken focus (e.g., exclude if the only chicken reference is in the name of the restaurant or retailer)
About retailer(s) that is/are NOT poultry specific

**Inclusion criteria**. *Any article related to COVID-19 and the chicken meat food system (with the terms poultry and chicken used interchangeably), including, but not limited to the following:*
Changes in market access
Changes in market shares
Economic vulnerability of value chain businesses
Closure of value chain businesses due to changes in demand
Change in business practices
Change in distribution mechanisms
Diversification of value chain businesses
Increasing vulnerability to other threat(s), e.g., other disease threats
Change in value chain management practices
Labour in value chain affected
Change in labour regulation
Infection of workers in value chain business
Closure of value chain business due to COVID19 infection in workers
Implementation of social distancing in value chain sites/business
Culling of (healthy) chickens due to workers' shortage
Culling of (healthy) chickens
Animal welfare
Vulnerability of workers in value chain
Change in supply chain structure
Coping strategies
