# Supplementary material for: A Global Media Analysis of the Impact of the COVID-19 Pandemic on Chicken Meat Food Systems: Key Vulnerabilities and Opportunities for Building Resilience
Source: Sustainability. Author manuscript; Available in PMC 2024 Dec 9. (PMC7617157; doi:10.3390/su13169435)
Supplement: Annex C [file EMS197569-supplement-Annex_C.pdf]

Annex C – Final themes and codes used for data extraction

In this table, **themes, subthemes** and **initial codes** were a result of the preliminary review and conceptualisation, whereas the full list of **topics** in the extraction table were the result of the information gathered and further grouped in **categories**. In "Categories", **undefined** refers to single topics with no clear category, n<5; **Other** includes unrelated/ungrouped topics n<10.

Theme	Sub-theme	Categories	Sub-categories/topics (extraction codes)	n
**DEMAND, MARKET EFFECTS**	**CHANGES IN CONSUMER DEMAND:** *Increase or decrease in demand for chicken / chicken meat, substitute or complement products*	Decreased	*Buying less*	59
			Buying less because of scare	239
			Buying less because fewer tourists	2
			Buying less because decreased income	3
			Effect of substitute products	20
		Increased	*Increased*	50
			increased home consumption	15
			Panic buying	70
			Buying more	5
			Buying take out food meals for entire family	2
		Other	*Large fluctuations*	3
			No change in demand	4
			Different cuts	1
	**REASONS FOR DEMAND CHANGE:** *Why the consumer demand changes, (-) decreased demand, (+) increased demand*	Beliefs, preferences	*Fear of transmission through chicken (-)*	235
			No fear of transmission anymore (+)	8
			Chicken as healthy food (+)	5
			Prefer non-perishable foods (-)	1
			African Swine Fever causing less pork (+)	1
			Alternative to red meat (+)	1
		Closures/restrictions consequences	*Lockdown - no access to market(-)*	13
			Curfew (-)/(+)	3
			Restaurant/hospitality sector/events closure (-)	57
			Fear of food shortage (+)	47
			Travel and gathering restrictions (-)	1
		Undefined	*Not stated/Unclear*	6
		Messages on safe chicken eating	*Messages on safe chicken eating*	73

		Chicken listed as healthy food during lockdown	*1*
**INFORMATION MANAGEMENT:** *Activities that can lead to change in behaviour*	Misinformation	*Misinformation*	*188*
		Messages on unsafe chicken eating	*14*
		Information about chickens that may create fear	*22*
		Information about chickens that may change behaviour	*11*
	Reassurance	*Government reassuring consumers about supply chain*	*7*
		Traders/suppliers reassuring consumers supplies are OK	*3*
	Other	*Use of social media or phones to engage consumers*	*3*
		Priming	*5*
		Who spreads the information	*4*
		Recommendations to reduce household food waste during lockdown	*2*
**PEOPLE AND THEIR RELATIONSHIP WITH CHICKEN / CHICKEN MEAT:** *How people see chicken, what role it has in their diet*	Chicken an essential product/staple	*Chicken an essential product/staple*	*25*
	Changes in consumer perceptions regarding animal food origin	*Changes in consumer perceptions regarding animal food origin*	*12*
	Chicken as pets	*Chicken as pets*	*8*
	Chicken as comfort food	*Chicken as comfort food*	*2*
**CHANGES IN CHICKEN or CHICKEN PRODUCT SALES:** *How chicken product sales change*	Increase	*Increase*	*66*
	Decrease/farm level decrease	*Decrease/farm level decrease*	*263*
	Other	*Different cuts sold*	*3*
		Returning to normal	*5*
		No change in sales	*3*
**CHANGES IN CHICKEN or CHICKEN MEAT PRICES**	Increase	*Retail level increase*	*63*
		Farm level increase	*5*
		Retail and farm increase	*4*
		Wholesale increase	*3*
		Overall increase	*6*
	Decrease	*Retail level decrease*	*88*
		Farm level decrease	*74*
		Retail and farm decrease	*37*
		Wholesale decrease	*4*
		Sub-national level decrease	*3*

			Overall decrease	*54*
			National level decrease	*35*
		No change	*No retail change*	*2*
			No change	*1*
		Other	*Retail increase, farm decrease*	*9*
			International market prices	*1*
			Returning to normal	*1*
**VALUE CHAIN EFFECTS**	**VALUE CHAIN BUSINESS CLOSURE:** *Closure of a chicken meat value chain entity because of the pandemic or associated mitigation measures*	Meat processing plants/ Packing plant closure	*Meat processing plants/ Packing plant closure*	*74*
		Restaurants	*Restaurants*	*49*
		Farms	*Farms*	*11*
		Retailers	*Retailers*	*33*
		Other	*Business sales and stock market losses*	*4*
			Schools, canteens	*1*
			Smallholders	*1*
	**SUPPLY CHAIN FUNCTIONING:** *how the supply chain functions*	Fragility or disruption	*Fragility*	*36*
			Disruption	*92*
		Increasing supply	*Oversupply*	*31*
			Increasing supply	*5*
		Shortage	*Shortage*	*64*
		Stability	*Stability*	*64*
	**LINKS TO OTHER CHAINS:** *Effects on other chains caused by changes in the chicken meat chain*	Feed	*Feed shortage*	*66*
			Feed price increases	*7*
			Feed price decreases	*25*
			Reduced demand for feed	*13*
			Reduction in feed sales and price	*1*
			Reduction in feed sales	*1*
		Substitute products	*Substitute products (e.g. vegetables or other meats)*	*18*
		Other	*Transport*	*9*
			Farming equipment trade loss	*2*
			Food processing equipment	*1*
			Taverns selling spirits	*1*

	**REQUESTS and POWER:** *What value chain actors demand (and get), what power they have and inequalities*	Request or demands for support	*Demands from stakeholders for protection*	*65*
			Request for social support	*6*
			Help request from industry (e.g. flexibility on inspection)	*13*
			Public sector intervention	*44*
		Inequalities and power unbalance	*Inequality in chain*	*7*
			Power of food industry	*3*
		Role of Unions	*Role of Unions*	*17*
		Undefined	*Consumers/other stakeholders to follow advice*	*1*
**TRADE**	**TRADE:** *How chicken meat trade is affected by the pandemic*	Reduced opportunity for international trade	*Loss*	*47*
			Import ban	*4*
			Exports ceased/decreased because of COVID19	*1*
			No imports because of COVID19	*1*
			Increased restrictions at ports	*1*
		Opportunity or change	*Opportunity*	*31*
			Increase	*3*
			National self-sufficiency	*2*
			Global meat business changing	*23*
			Change in export duties	*2*
		No change	*No change*	*4*
**LABOUR and LIVELIHOODS**	**LABOUR AFFECTED IN VC BUSINESS:** *How the pandemic affects labour in the chicken value chain*	Labourers with COVID19 and risks	*Labourers with COVID19 in poultry plants*	*118*
			Difficulty in implementing social distancing	*15*
			Potential risks for food chain workers	*3*
		Job loss / unemployment	*Job loss / unemployment*	*20*
		Labour shortage	*Labour shortage*	*38*
		Rights	*Labour rights*	*49*
			Meat and poultry inspector rights	*1*
			Labourers with COVID19 in poultry plants/Labourers refusing to work/Union actions	*1*
			Labourers refusing to work due to conditions	*2*
			Labourers asking for better working conditions	*1*
	**ECONOMIC HARDSHIP**	Economic hardship	*Loss of income*	*64*
			Loss of livelihood	*24*

			Business going bust	*5*
**MITIGATION AND INTERVENTIONS**	**ECONOMIC RELIEF/SUPPORT:** *What is provided by government or others to help the businesses that are struggling*	Regulating value chain from outside and inside	*Cap on prices/Prices stabilisation*	*8*
			Marketing support from Government	*2*
			Chicken stated as an essential product	*4*
			Support for employees	*6*
			Cash donation by chicken association	*1*
		Support measures from authorities or industry	*Subsidies*	*12*
			Damage control plans	*9*
			Loan schemes	*16*
			Credit	*3*
			Fiscal and tax support	*1*
			Demand for economic support	*2*
			Relief package (not specified)	*1*
		Other	*Criminal investigation*	*2*
			Other	*20*
	**FOOD DONATION:** *Giving away chicken to those in need*	Food donation	*Food donation*	*111*
		Food aid	*Food aid*	*19*
	**EFFORTS TO NORMALISE MARKET DYNAMICS:** *Measures implemented to bring market dynamics back to a more "normal" situation*	Industry changes for resilience/survival	*Stockpile inputs*	*5*
			Connect small and large farmers/processors	*2*
			Move sales online	*1*
			Use meat in public service (e.g. school meals)	*2*
			Transfer produce to other retail channels	*2*
			stockpile poultry	*1*
		Addressing consumer response	*Diminish/address consumer fears*	*150*
			Facilitate social distancing in retail	*9*
			Government and/or other authorities/bodies correcting/refuting misinformation	*28*
		Control and regulation of supply. Top-down approaches	*Regulate stockpiling*	*15*
			Price controls	*10*
			Regulated supply quotas	*3*
			Coordinate supply/monitor prices	*1*
			Allow farmers to access their farms	*5*
			Allow input transport	*6*

			Government support for new supply chain arrangements	*6*
			Declare meat production essential activity or facilitate supply	*12*
			Industry efforts to increase supply	*10*
			Promote re-opening of markets	*3*
			Keep value chain businesses open	*1*
			Government policies and resources to increase production	*2*
			Lift movement restrictions	*2*
	**OUTBREAK MANAGEMENT**	Closures and restrictions	*Market closures*	*9*
			Bans	*18*
			Limit access	*3*
			Cross-borders control	*7*
			Transport	*6*
			Packing plant closure	*1*
			curfew	*3*
			Restaurant closure	*5*
			travel and gathering restrictions	*1*
		Enable prevention, control or mitigation measures	*Distancing, test and trace for value chain actors*	*17*
			PPE and sanitising	*14*
			Animal testing	*1*
			Organise alternative distribution/retail systems	*2*
			Supply stabilisation programmes	*1*
		Information provision	*Travel advice*	*4*
			Behaviour advice	*12*
		Prosecution	*Prosecution*	*12*
		Undefined	*Free chicken for low case numbers*	*1*
		Various	*Various*	*8*
**COPING**	**COPING CONSUMERS:** *Strategies consumers use during pandemic*	Keeping more chickens	*Keep more chickens*	*24*
			Keeping backyard hens	*9*
		Eat different foods	*Eat different foods*	*10*
		Stockpiling	*Stockpiling*	*12*
	**COPING PROCESSORS:** *Strategies*	Business operations	*Direct to consumer sales*	*8*
			Lower prices	*3*
			Altering production	*27*

processors use during pandemic		*Cold chain support*	*1*
		Efforts to increase supply (e.g. production)	*12*
		Production change to different cuts	*3*
		Increasing hiring	*2*
		Digitalisation and online services	*3*
	Prevention/biosecurity	*Distancing, test and trace for value chain actors*	*1*
		Distancing	*1*
		Distancing, event cancellation	*1*
		Sanitation	*3*
		Sanitation/distancing	*1*
		Temperature test	*1*
		PPE/testing etc. at processing plants	*23*
	Various strategies	*Mixed: Altering production/distancing/sanitation/employment*	*2*
**COPING RETAILERS AND RESTAURANTS:** *Strategies retailers and restaurants use during pandemic*	Business operations	*Diversification*	*19*
		Different delivery	*45*
		Offers	*13*
		Different marketing	*10*
		Changing suppliers	*2*
		Simplification	*11*
		Using locally available ingredients	*1*
		Using safer ingredients (dried, frozen, pasteurised)	*1*
		Closing and/or changing menu to fish, mutton etc.	*1*
	Prevention/biosecurity	*Distancing*	*3*
		Sanitation/distancing/home delivery	*4*
		Simplification/distancing	*1*
		Sanitation/distancing	*1*
	Undefined	*Purchase limits*	*1*
	Various strategies	*Various*	*4*
**COPING FARMERS:** *Strategies farmers use during pandemic*	Reduce costs	*Get rid of birds*	*71*
		Sell or break hatching eggs	*3*
		Give birds away for free	*5*
		Reduce production costs	*10*
	Measures to survive	*Find other labour*	*2*

			Request for correct messaging	*2*
			Request compensation/subsidies	*8*
			Lower prices	*7*
			Diversification	*4*
			Direct sales	*11*
			Sanitation/distancing	*2*
			Contingency planning	*1*
		Various strategies	*Various*	*11*
**EPIDEMIOLOGICAL FACTORS**	**EPIDEMIOLOGICAL INFORMATION RELEVANT TO CHICKEN MEAT FOOD SYSTEMS**	Involvement of live bird markets	*Involvement of live bird markets*	*28*
		Transmission	*Transmission*	*41*
		Role of poultry farming	*Role of poultry farming*	*6*
		Poultry processors' role	*Poultry processors' role*	*11*
**OTHER**	**ANIMAL WELFARE:** *How COVID19 or associated dynamics affect animal welfare*	System failure	*Current production practices*	*6*
			Failure of "just in time system"	*2*
			no access to animal health services	*3*
			distribution issues	*1*
		Starvation	*Chickens starve*	*12*
			cannot feed animals	*9*
		Consequences: slaughtered/dumped	*Birds dumped alive*	*8*
			Birds buried alive	*27*
			Mass slaughter	*5*
		Other	*Change in slaughter technique*	*8*
			Importance	*5*
			Panic buying baby chicks	*3*
